# Decoding the Enigma of Gallbladder Perforation: Exploring Patient Profiles, Diagnosis, and Step-Up Management Pathways

**DOI:** 10.7759/cureus.84791

**Published:** 2025-05-25

**Authors:** Harpyar Singh, Raj Kumar Chejara, Nirupendra G Thippeswamy, Lirangla Sangtam, Eshan Kapoor, Mohhammad Zahid, Kuozokhotuo Suohu, Rajat Dinkar, Khushal Jindal

**Affiliations:** 1 Department of General Surgery, Vardhman Mahavir Medical College and Safdarjung Hospital, New Delhi, IND

**Keywords:** cholecystostomy tube, gall bladder diseases and gallstones, gallbladder diseases and gallstones, gallbladder perforation, spontaneous gallbladder rupture, step-up approach

## Abstract

Background and aim: Gallbladder perforation (GBP) is a serious, life-threatening condition that can result from acute cholecystitis, trauma, or gallstone disease. Diagnosis remains challenging due to its nonspecific symptoms, often mimicking other abdominal pathologies. This study aimed to investigate the clinical features, diagnostic approaches, management strategies, and outcomes of GBP at a tertiary care center.

Methods: A retrospective cohort study was conducted at Vardhman Mahavir Medical College (VMMC) and Safdarjung Hospital from January 2022 to February 2025. Patients aged 18 years and older diagnosed with GBP were included, with those with trauma or malignancy excluded. Demographic, clinical, diagnostic, and treatment data were collected and analyzed.

Results: A total of 72 patients were included, predominantly aged >40 years (73.9%), with a higher prevalence in females (67.1%). Diabetes (26.1%) and hypertension (17.8%) were the most common comorbidities. The most frequent symptom was right upper quadrant pain (93%), with a mean presentation time of 18.6 days. Imaging (USG and CT) revealed that 67/72 cases were diagnosed with GBP, with the fundus being the most common site of perforation. Most patients (69.8%) received conservative management with antibiotics, while 16.6% underwent percutaneous drainage procedures. The overall mortality rate was 2.7%.

Conclusions: GBP presents with diverse clinical manifestations, and early diagnosis is crucial to reduce complications. Non-resolution of symptoms with significant pericholecystic collection warrants percutaneous drainage, a minimally invasive procedure with low morbidity. A multidisciplinary approach is essential for optimal management, particularly in complex cases requiring interval cholecystectomy.

## Introduction

Gallbladder perforation (GBP) is a potentially life-threatening condition that can significantly complicate the clinical course of patients with gallstone disease. It is a rare complication that may arise from various causes, including gallstone disease, trauma, gallbladder malignancy, or acalculous cholecystitis. Despite advancements in imaging technologies, diagnosing GBP remains a challenge due to its nonspecific clinical presentations, which often mimic other abdominal pathologies, such as acute cholecystitis, liver abscesses, pancreatitis, and others [1].

Historically, in 1934, Niemeier categorized GBP into the following three types: type I (acute free perforation into the peritoneal cavity), type II (subacute perforation with a pericholecystic abscess), and type III (chronic perforation resulting in a cholecystoenteric fistula) [2]. Anderson and Nazem and Ibrarullah et al., in independent case reports, reported instances of cholecystobiliary fistulae and introduced a "type IV" perforation classification [3,4]. Both Anderson and Nazem and Ibrarullah et al. compared cholecystobiliary fistulas to Mirizzi's type II syndrome, which was first described in 1948 and later modified by McSherry et al. in 1982 into a two-stage classification based on findings from endoscopic retrograde cholangiopancreatography (ERCP) and percutaneous transhepatic cholangiography [5,6]. In contrast, Kochar et al. suggested consolidating various fistulas, such as cholecystobiliary, cholecystocutaneous, cholecystoenteric, and cholecystohepatic, into a single category of "type III perforation" to maintain consistency in reporting [7]. Despite the inconsistencies in GBP classification, we have adopted Niemeier's original classification in this study to avoid confusion. Understanding these types is crucial for determining the appropriate management approach, which may range from conservative management to urgent surgical intervention.

Given the severity and potential complications associated with GBP, including high morbidity and mortality rates, a thorough investigation into patient profiles, diagnostic pathways, and management strategies is essential. This study aimed to bridge the existing knowledge gap by providing comprehensive insights into the epidemiology, diagnosis, treatment, and outcomes of GBP in a high-volume tertiary care teaching hospital in India.

## Materials and methods

Study design, population, and sampling

This retrospective cohort study was conducted on patients diagnosed with gallbladder perforation (GBP) at Vardhman Mahavir Medical College and Safdarjung Hospital over four years, from January 20221 to February 2025. The study included all patients aged 18 years and older diagnosed with GBP, confirmed through imaging or surgical findings. Patients whose perforations were secondary to trauma or malignancy were excluded from the study, as were those with incomplete medical records.

Data collection and statistical analysis

Data were systematically gathered using a structured proforma, which included detailed information on patient demographics, clinical symptoms, type of perforation, diagnostic modalities, management strategies, and outcomes. Descriptive statistics were employed to summarize the patient demographics, clinical characteristics, and diagnostic findings. Frequencies and percentages were calculated for categorical variables, such as age groups, gender, and types of gallbladder perforations. Measures of central tendency (mean, median) and dispersion (standard deviation) were used to describe continuous variables, including the length of hospital stay and time to diagnosis. These descriptive statistics provided a comprehensive overview of the patient population and the distribution of clinical outcomes, assisting in identifying trends and patterns relevant to management strategies.

## Results

Demographic and clinical characteristics

The study cohort primarily comprised individuals aged over 40 years, representing 54 patients (73.9%). The highest prevalence was observed in the 40-60-year age group, which included 35 individuals (47.9%). The mean age at presentation was 58.2 years. A significantly higher prevalence was noted among females (n=49, 67.1%). Diabetes mellitus was the most common comorbidity, identified in 19 patients (26.1%), followed by hypertension, which was observed in 13 individuals (17.8%). Notably, eight patients (11.1%) had both diabetes mellitus and hypertension. Gallstone disease was present in 59 patients (81.9%), with the majority having gallstones larger than 10 mm in diameter (Table 1).

**Table 1:** Demographic and clinical characteristics. DM: diabetes mellitus

Age	
<40 years	19 (26.3%)
40-60 years	35 (48.6%)
60-80 years	16 (22.2%)
>80 years	3 (4.16%)
Gender	
Male	24 (33.3%)
Female	49 (68%)
Comorbidities	
Hypertension	13 (19.1%)
Diabetes mellitus	19 (26.3%)
HTN+DM	8 (11.1%)
Gallstone disease	59 (81.9%)

Clinical presentation

The most frequently reported presenting symptom was right upper quadrant (RUQ) pain, documented in 67 cases (93%). Fever was observed in 13 patients (18%). A RUQ lump was identified in only three patients, while an additional three presented with jaundice concomitant with RUQ pain. Two patients exhibited features of intestinal obstruction secondary to gallstone ileus. The mean interval between symptom onset and clinical presentation was 18.6 days. Notably, 32 patients (52.7%) presented after a duration exceeding three weeks, whereas 20 patients (27.7%) sought medical attention within three days of symptom onset. A total of 31 patients (43%) presented to the surgical emergency department with acute to subacute RUQ pain, while the remaining patients presented to the outpatient department with vague or nonspecific RUQ discomfort (Table 2). At the time of presentation, tachycardia (defined as a heart rate exceeding 100 beats per minute) was observed in 32 patients (44.4%). Leukocytosis (total leukocyte count {TLC} >11,000/mm³) was observed in 25 patients (34.7%), with nine (12.5%) exhibiting a TLC count exceeding 20,000/mm³. Among these patients with TLC >20,000/mm^3^, four presented with diffuse abdominal pain. Bilirubin levels were normal in 54 patients (75%), while 18 patients (25%) had serum bilirubin levels >2 mg/dL, including two patients with bilirubin levels >10 mg/dL. Thirty-eight patients (49%) had alkaline phosphatase (ALP) levels >200 IU/L, with 25 individuals (34.7%) presenting with normal ALP levels (Table 3).

**Table 2:** Clinical presentation variables of gallbladder perforation patients. RUQ: right upper quadrant

Clinical presentation	n (%)
Symptoms	
RUQ pain	67 (93%)
Diffuse pain abdomen	6 (8.3%)
Fever	13 (18%)
Palpable mass	3 (4.1%)
Jaundice	3 (4.1%)
Pain RUQ + fever + palpable RUQ mass	3 (4.1%)
RUQ pain + fever	9 (12.5%)
Obstruction	2 (2.7%)
Duration between onset and presentation	
<3 days	20 (27.7%)
3-7 days	11 (15.2%)
1-3 weeks	9 (12.5%)
>3 weeks	32 (44.4%)
Heart rate	
>100 beats/min	32 (44.4%)
<100 beats/min	50 (69.4%)
Modified Neimer’s type	
Type I	22 (30.5%)
Type II	40 (55.5%)
Type III	10 (13.8%)

**Table 3:** Biochemical parameters of gallbladder perforation patients. Normal lab reference ranges: total leukocyte count = 4.0-10.0 x 10^3^/mm^3^, total bilirubin = 0.30-1.20 mg/dL, and ALP = 40-128 U/L. TLC: total leukocyte count; ALP: alkaline phosphatase

Biochemical investigations		n (%)
TLC	4-11 x 10^3^/mm^3^	47 (65.2%)
	11-20 x 10^3^/mm^3^	16 (22.2%)
	>20 x 10^3^/mm^3^	9 (12.5%)
Total bilirubin	<2 mg/dL	54 (75%)
	2-5 mg/dL	10 (13.8%)
	5-10 mg/dL	7 (9.7%)
	>10 mg/dL	2 (2.7%)
ALP	<140 U/L	25 (43.7%)
	140-200 U/L	19 (26.3%)
	>200 U/L	38 (52.7%)

Imaging findings

**Figure 1:**
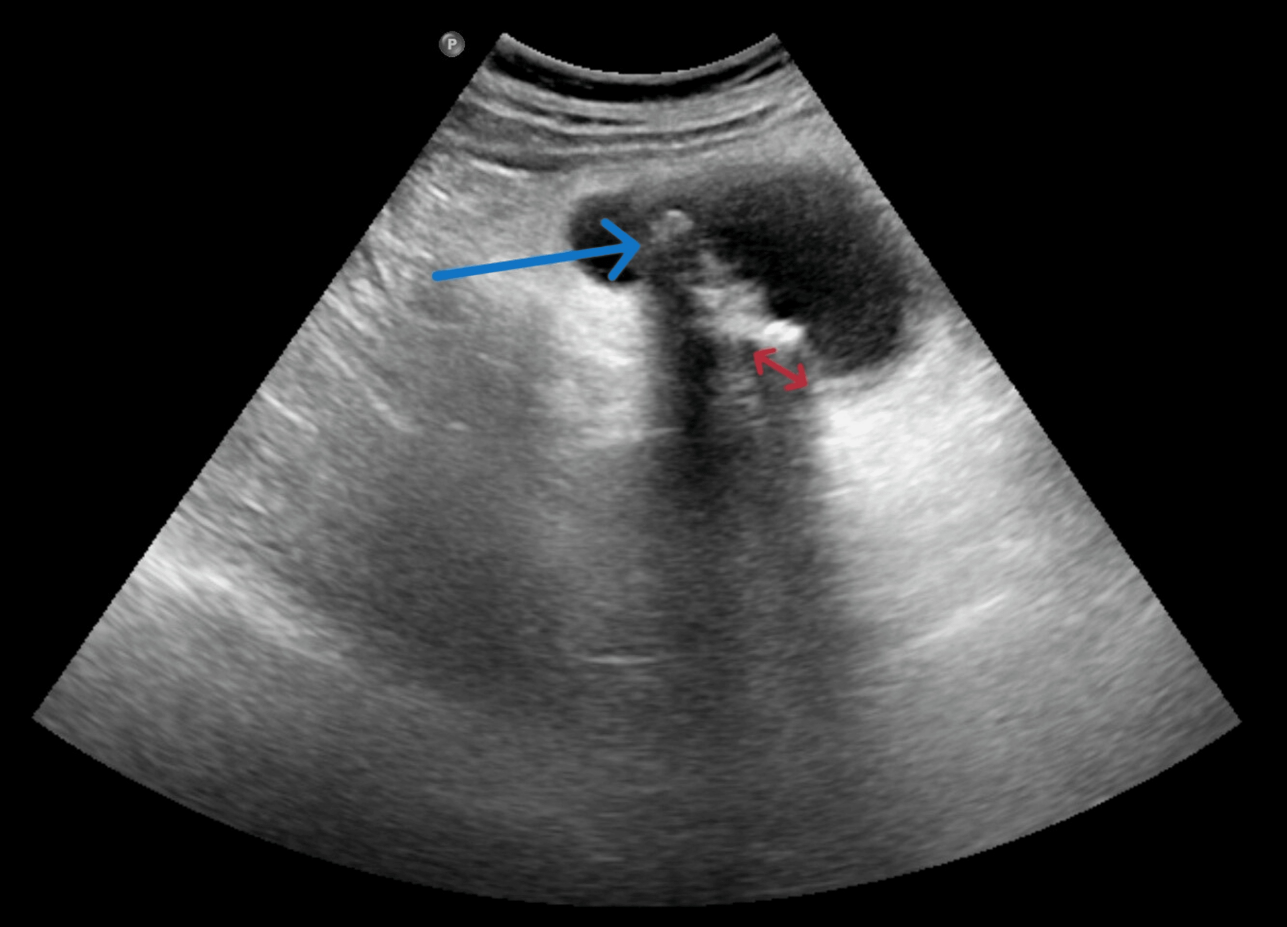
USG view of gallbladder perforation. The blue arrow represents a gallstone with posterior acoustic shadow and the red arrows represent rent in the gallbladder wall.

Gallbladder perforation (GBP) was diagnosed preoperatively using ultrasonography (USG) in 44 out of 72 cases, corresponding to a sensitivity of 61.1%. The principal diagnostic features included pericholecystic fluid collection in conjunction with a discernible defect in the gallbladder wall, which was identified in 39 cases (Figure 1). Missed diagnoses predominantly involved type III GBP, such as cholecystoenteric and cholecystobiliary fistula, which was diagnosed intraoperatively, and also included cases presenting with gallstone ileus. In one of the patients presenting with acute abdominal pain, the abdomen X-ray chest showed free air under the right dome of the diaphragm. In six patients who underwent elective surgery for symptomatic cholelithiasis with USG revealing the wall-echo-shadow (WES) complex, cholecystoenteric fistula was found intraoperatively. In patients with suspected cholecystoenteric communications during the preoperative workup, contrast-enhanced computed tomography (CECT) of the abdomen and magnetic resonance cholangiopancreatography (MRCP) were employed to confirm the diagnosis and assess the underlying etiology.

In 52 cases (72%), the gallbladder wall thickness exceeded 5 mm. Among these, nine cases demonstrated irregular or focal wall thickening. Further evaluation revealed that seven of these nine cases were found to be gallbladder carcinoma, and these were subsequently excluded from the study. The remaining two cases represented focal gallbladder perforations mimicking mass lesions. The fundus was identified as the most frequent site of perforation, observed in 47 patients (65.2%). Intrahepatic collections consistent with intrahepatic gallbladder perforation were identified in six patients (8.3%). Gallstones were present in 59 cases (81.9%), with 50 patients (69.4%) harboring calculi greater than 10 mm in diameter, and 29 patients (40.2%) with stones exceeding 20 mm (Table 4).

**Table 4:** Imaging features. CECT: contrast-enhanced computed tomography; CBD: common bile duct; GB: gallbladder

Gallbladder characteristics		n (%)
USG/CECT abdomen/intraoperative findings		
Wall thickening	<5 mm	30 (41.6%)
	>5 mm	42 (58.4%)
	Focal/irregular	9 (12.5%)
Site of perforation	Anterior wall	12 (16.6%)
	Posterior wall	0
	Fundus	47 (65.2%)
	Not specified	13 (18%)
Associated findings	Intrahepatic collection	6 (8.3%)
	Cholelithiasis	59 (81.9%)
Size of calculi	<10 mm	9 (12.5%)
	10-20 mm	21 (29.1%)
	>20 mm	29 (40.2%)
Signs of pancreatitis		6 (8.3%)
Mirizzi syndrome		3 (4.1%)
Dilated CBD		4 (5.5%)
Choledocholithiasis		2 (2.7%)
Evidence of GB cancer metastasis	Hepatic lesions	4 (5.55%)
	Adjacent lymphadenopathy	6 (8.3%)
	Ascites	7 (9.7%)
Signs of obstruction	Gallstone ileus	2 (2.7%)
Rare findings	Splenic artery aneurysm	1 (1.3%)
	Cholecystocolic fistula	2 (2.7%)

**Figure 2:**
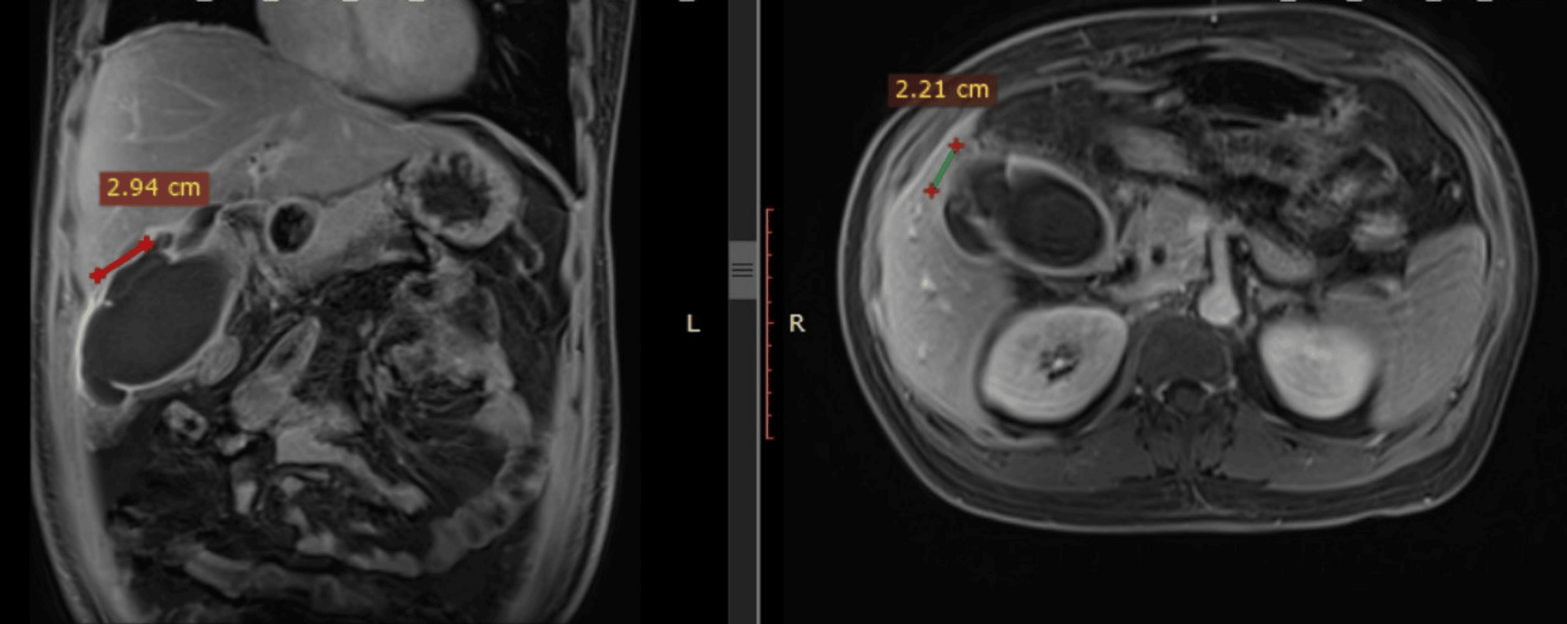
Magnetic resonance cholangiopancreatography (MRCP) showing rent in gallbladder wall (red crosses) with pericholecystic collection.

Peripancreatic collections and enlargement of the pancreatic head were observed in six cases (8.3%), suggestive of pancreatitis as a complication of GBP. In these cases, the diagnosis was further supported by elevated serum amylase and lipase levels. In three cases, large gallstones were impacted at the gallbladder neck, exerting pressure on the common hepatic duct, consistent with Mirizzi syndrome. A dilated common bile duct (CBD) was noted in four cases, with choledocholithiasis confirmed in two of them. Magnetic resonance cholangiopancreatography (MRCP) was utilized in all cases with CBD dilatation (Figure 2). Two patients presenting with signs of acute bowel obstruction underwent CECT of the abdomen, which revealed intraluminal gallstones. These patients underwent exploratory laparotomy with enterotomy for gallstone retrieval. Further evaluation was planned to address the underlying cholecystoduodenal fistula in a staged manner. One of the patients had concurrent gallstone-induced acute pancreatitis with gallbladder perforation and was diagnosed with a splenic artery aneurysm as a local complication of acute pancreatitis on CECT of the abdomen. Splenic artery aneurysm was successfully managed via endovascular coiling performed by the interventional radiology team.

Additionally, two patients with no preoperative indication of GBP, who underwent laparoscopic cholecystectomy for symptomatic cholelithiasis, were intraoperatively found to have cholecystocolic fistulae. Both patients underwent cholecystectomy along with repair of the colonic defect. One of these cases required conversion to open surgery. The other patient developed a postoperative bile leak on the first postoperative day, which was effectively managed with endoscopic retrograde cholangiopancreatography (ERCP) and biliary stenting.

Modified Niemeier’s classification

Based on clinical, radiological, and intraoperative findings, the most common type of gallbladder perforation was Niemeier’s type II (n=40, 55.5%) most commonly diagnosed by ultrasound, followed by type I (n=22, 30.5%) most commonly diagnosed on emergency exploratory laparotomy, and type III (n=10, 13.8%) diagnosed most commonly intraoperatively during elective laparoscopic cholecystectomy.

Surgical interventions

**Figure 3:**
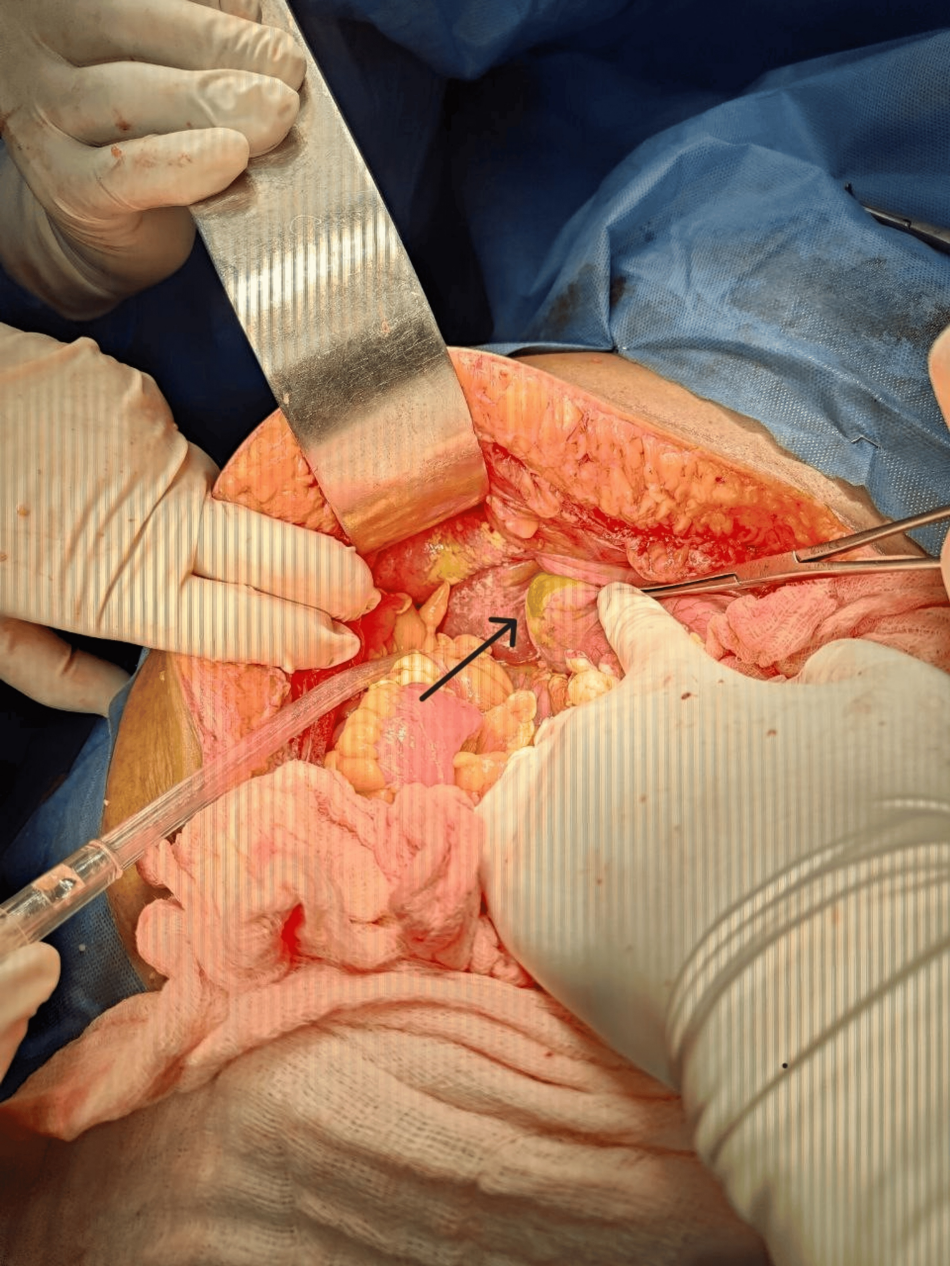
The site of perforated gallbladder during exploratory laparotomy (arrow).

Six patients (8.3%) underwent emergency exploratory laparotomy with cholecystectomy for diffuse abdominal pain and tenderness, supported by ultrasound findings of gallbladder perforation (type I gallbladder perforation) (Figure 3). In two of these cases, Calot’s dissection was not possible, necessitating subtotal cholecystectomy with a fundus-first approach. Fifty patients (69.8%) underwent conservative management with broad-spectrum antibiotics and serial monitoring. For patients with significant pericholecystic collection, persistent leukocytosis, and tachycardia, ultrasound-guided percutaneous drainage was performed in 12 cases (16.6%). All patients in the conservative management group showed resolution of symptoms and were scheduled for interval cholecystectomy, except for one 66-year-old female, whose sepsis did not resolve despite percutaneous drainage. She ultimately underwent emergency exploratory laparotomy with peritoneal lavage and tube cholecystectomy. The patient expired on postoperative day two.

Laparoscopic cholecystectomy and conversion

**Figure 4:**
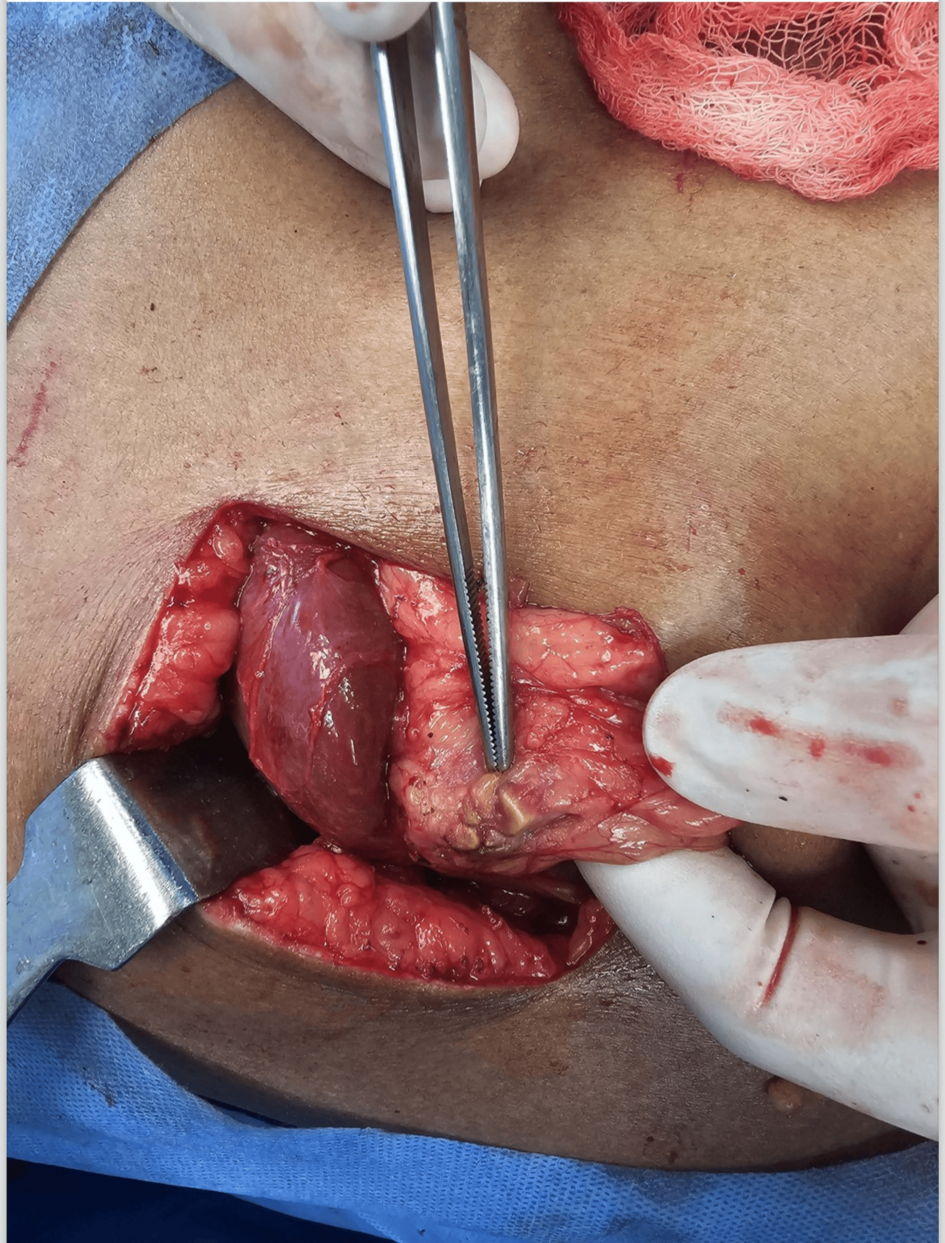
Forceps point gallstones extruding through the gallbladder wall during an open cholecystectomy.

Interval laparoscopic cholecystectomy was performed in 48 patients, and there was a laparoscopy-to-open conversion in four cases due to dense adhesions and technical difficulties. Six patients underwent elective open cholecystectomy in anticipation of frozen Calot's anatomy, and four of these cases had a subtotal cholecystectomy as a bailout procedure (Figure 4).

Mortality and complications

The mortality rate in this cohort was 2/72 (2.7%), with two deaths occurring after exploratory laparotomy. Procedure-related complications included postoperative bile leaks in two cases (both involving CBD injury) and persistent gallbladder fossa collection, which led to prolonged hospital stays (>7 days) in four patients. Gallbladder perforation-related complications included biliary peritonitis, sepsis, multiple organ dysfunction syndrome (MODS), pancreatitis, hepatic abscess, and splenic artery aneurysm (Table 5). The patient with a splenic artery aneurysm underwent interventional radiology-guided coiling.

**Table 5:** Management and outcome of gallbladder perforation. MODS: multiple organ dysfunction syndrome; GB: gallbladder

Management	n (%)
Conservative management	50 (69.4%)
Percutaneous drainage procedure	12 (16.6%)
Emergency exploratory laparotomy with cholecystectomy	6 (8.3%)
Emergency exploratory laparotomy with enterotomy for gallstone ileus	2 (2.7%)
Interval cholecystectomy	
Laparoscopic cholecystectomy	48 (66.6%)
Open cholecystectomy	6 (8.3%)
Lap to open cholecystectomy	4 (5.5%)
Outcomes	
Mortality	2 (2.7%)
Complications	
Procedure related	
Postoperative bile leak	2 (2.7%)
GB fossa collection with prolonged stay (7 days)	4 (5.5%)
GB perforation related	
Biliary peritonitis	6 (8.3%)
Hepatic abscess	8 (11.1%)
Pancreatitis	6 (8.3%)
Splenic artery aneurysm	1 (1.3%)
Sepsis	6 (8.3%)
MODS	3 (4.1%)

## Discussion

This study underscores that gallbladder perforation (GBP) is more frequently encountered in individuals over the age of 40 years, with a predominance among female patients. Comorbidities, particularly diabetes mellitus (26.1%) and hypertension (17.8%), were common, with 11.1% of patients presenting with both conditions. The most prominent clinical manifestation was right upper quadrant (RUQ) pain, often accompanied by localized or generalized tenderness. Previous literature has suggested that high fever and leukocytosis are not reliable diagnostic indicators of GBP, as these findings are present in only a subset of patients [7]. Consistent with this, fever was observed in just 13 patients (18%) in our cohort, while leukocytosis was documented in 25 patients (34.7%). Based on these findings, GBP should be regarded as a critical differential diagnosis in patients aged 40 years and above, particularly those with comorbidities such as diabetes mellitus and hypertension presenting with RUQ pain.

The mean age at presentation in our cohort was 58.2 years, aligning closely with the mean age of 62.1 years reported in major systematic reviews [8]. The strong association between GBP and comorbid conditions, notably diabetes and hypertension, reinforces the hypothesis that ischemic changes - exacerbated by advancing age and cystic duct obstruction from large gallstones - play a key role in the pathogenesis of GBP. In this study, 81.9% of patients had cholelithiasis, with more than 85% of the calculi measuring over 10 mm in diameter. These findings are comparable to those reported by Gupta et al., who documented an incidence of cholelithiasis of 84.8% in a similar patient population [9]. The fundus was identified as the most frequent site of perforation (n=47; 65.2%), which is attributable to its higher susceptibility to ischemia. Similar findings were reported by Derici et al., who noted that the fundus was involved in 60% of cases [10]. These data support the theory that the pathophysiology of GBP involves ischemic injury facilitated by underlying comorbidities, compounded by mechanical obstruction caused by large gallstones.

In a national German quality assurance database comprising over 45,000 patients with acute cholecystitis, the incidence of acute perforated cholecystitis was reported to be 9.7% [11]. Recent years have seen a declining trend in the incidence of type II GBP, a change that may be attributed to revisions in the indications for cholecystectomy under the Tokyo Guidelines, which now advocate for more aggressive surgical management in selected cases of acute cholecystitis [12]. The clinical presentation of GBP remains heterogeneous, ranging from acute diffuse abdominal pain to chronic, vague RUQ discomfort, and, in some instances, intestinal obstruction. This variability complicates timely diagnosis and management. Notably, over half of the patients in our study presented more than three weeks following symptom onset, suggesting that early manifestations of GBP may be subtle, underrecognized, or neglected. Accordingly, in patients with known risk factors who present with vague or subacute RUQ pain, prompt diagnostic evaluation is imperative.

Ultrasonography continues to be an effective diagnostic tool for GBP, with a sensitivity of 93% in the present study. In a related study by Sood et al., sonographic evidence of gallbladder wall defects and perforation was observed in 16 of 23 patients (70%) [13]. In selected cases, particularly those with suspected complications such as cholecystoenteric fistulae, gallstone ileus, or other complex biliary pathologies, additional imaging modalities such as contrast-enhanced computed tomography (CECT) and magnetic resonance cholangiopancreatography (MRCP) are recommended. Plain chest radiography in the erect position revealed free air under the right hemidiaphragm in only one patient, reinforcing the notion that pneumoperitoneum is a rare radiographic finding in cases of GBP.

**Figure 5:**
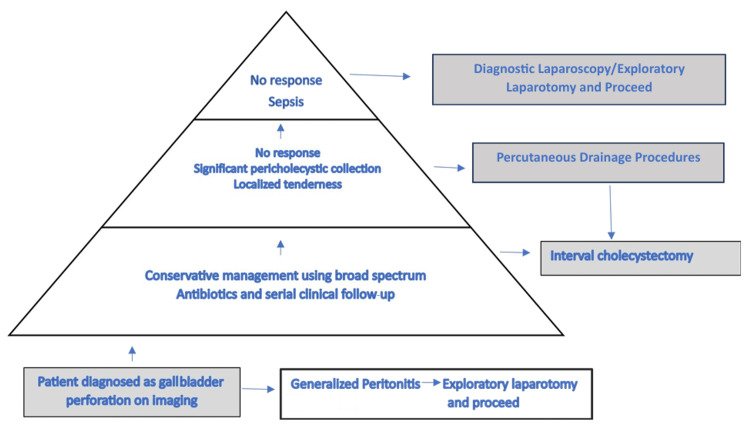
Step-up management pathway for management of gallbladder perforation. The image is created by the author (Harpyar Singh) of this study.

The management of GBP necessitates a multidisciplinary, step-up approach, as illustrated in Figure 5. For patients with Niemeier type I GBP, management is relatively straightforward, with most requiring exploratory laparotomy and cholecystectomy. However, a significant subset of our cohort (69.8%) was managed conservatively with broad-spectrum antibiotics, coupled with serial clinical and laboratory monitoring. In cases where symptoms persist or where significant pericholecystic collections and localized peritonitis are identified, image-guided percutaneous drainage becomes necessary. These minimally invasive interventions have demonstrated comparable outcomes to open surgical drainage and cholecystostomy, with reduced morbidity [14].

In cases where clinical symptoms persist despite conservative management and there is ongoing evidence of sepsis, surgical intervention via exploratory laparotomy should be considered. Surgical options include subhepatic drain placement, tube cholecystostomy, or cholecystectomy, depending on the intraoperative findings and the patient's clinical status. In the present cohort, the mortality rate was 2.7%, which is notably lower than the rates of up to 20% reported in prior literature on gallbladder perforation [15]. Specifically, the incidence and mortality associated with type II GBP arising from acute or chronic cholecystitis have been reported to be approximately 1.6% and 4.0%, respectively [16]. The relatively lower mortality observed in this study may be attributed to a multidisciplinary management approach, improved interventional radiology techniques, and the deliberate exclusion of cases where gallbladder malignancy was identified as the underlying cause of perforation.

For patients requiring interval cholecystectomy, both open and laparoscopic approaches remain viable and should be selected based on the surgeon's expertise and institutional resources. Previous reports indicate no statistically significant differences in postoperative complication rates between open and laparoscopic drainage or between early and delayed surgical intervention for type II GBP [17]. Nevertheless, both techniques pose distinct technical challenges. In the current study, laparoscopy-to-open conversion was necessary in four patients (5.5%), primarily due to dense adhesions encountered at Calot’s triangle, which also contributed to a heightened risk of postoperative bile leaks. Bailout procedures, including subtotal cholecystectomy, were employed in four patients (5.5%) and proved to be critical in managing technically difficult cases. Subtotal cholecystectomy serves as a valuable rescue strategy in situations where complete cholecystectomy carries substantial risk, offering comparable safety profiles while reducing the likelihood of bile duct injury [18,19]. These findings support the recommendation that interval cholecystectomy following GBP should ideally be performed at high-volume centers by experienced laparoscopic surgeons with access to advanced intraoperative and postoperative care.

Limitations

The primary limitation of this study is its retrospective design and single-center setting, which may constrain the generalizability of the findings. Additionally, data pertaining to the use of inflammatory biomarkers, such as procalcitonin and C-reactive protein (CRP), to guide decisions regarding percutaneous drainage were unavailable. Furthermore, the relatively small sample size limits the strength of the conclusions that can be drawn, particularly in the context of establishing standardized management protocols for gallbladder perforation.

## Conclusions

Gallbladder perforation (GBP) should be considered a high-priority differential diagnosis in patients presenting with vague right upper quadrant pain, particularly in individuals over the age of 40 years with underlying comorbidities such as diabetes mellitus and hypertension, especially in the presence of a history of gallstone disease. Type II GBP should be suspected in cases of chronic calculous cholecystitis with ultrasound findings demonstrating a wall-echo-shadow (WES) complex, and such patients should undergo further evaluation with magnetic resonance cholangiopancreatography (MRCP) and be referred to high-volume centers with experienced laparoscopic surgeons. Given the technical complexity associated with interval cholecystectomy following GBP, these procedures should ideally be undertaken at specialized centers by surgeons with extensive laparoscopic expertise. In patients presenting with localized tenderness due to GBP, a multidisciplinary team approach should be employed in accordance with the step-up strategy. Future prospective studies involving larger sample sizes and multi-institutional collaboration are warranted to further delineate optimal diagnostic and therapeutic strategies for this high-risk patient population and to validate the efficacy of the step-up approach in the management of GBP.
